# Estimating racial health disparities among adverse birth outcomes as deviations from the population rates

**DOI:** 10.1186/s12884-020-2847-9

**Published:** 2020-03-12

**Authors:** James A. Thompson, Melissa A. Suter

**Affiliations:** 1 College of Veterinary Medicine and Biomedical Science, Texas A&M University, College Station, TX 77843-4475 USA; 2 Department of Obstetrics and Gynecology, Baylor College of Medicine, 1 Baylor Plaza, Houston, TX 77030 USA

**Keywords:** Bayesian, Sum-to-zero, Maternal, Neonatal, Racial health disparity, Adverse birth outcomes

## Abstract

**Background:**

Despite significant research, the reasons for racial health disparities among adverse birth outcomes (ABO) remain largely unknown. The bulk of research into racial health disparities among ABO in the United States has concentrated on the risk of race and ethnic groups relative to the specific sub-population of non-Hispanic white women and their children. The objective of this study was to estimate the racial and ethnic risks among a set of neonatal and maternal health disparities while minimizing bias attributable to how the baseline risk was established.

**Methods:**

All birth records were obtained from the United States Natality database for the years 2014 to 2017. A Bayesian modeling approach was used to estimate the risk disparity for disorders by race. The estimation of the race-specific risks used a sum-to-zero constraint for the race regression coefficients.

**Results:**

Estimating racial health disparities relative to the overall population rate yielded novel results and identified perinatal health disparities for all the race groups studied.

**Conclusions:**

Unbiased risk estimates for racial disparities among ABO are now available for stimulating and initiating more complex causal modeling that can lead to understanding how racial health disparities for ABO are mediated and how they can be prevented.

## Background

In 2017, the United States infant mortality rate (IMR) was 5.8 deaths per 1000 live infant births and ranked an abysmal number 33 out of the 36 countries evaluated [1]. Even more concerning than the overall IMR is that non-Hispanic black infants had 2.4 times the rate of infant mortality compared to non-Hispanic white infants [2]. The IMR provides a valuable summary for both maternal and child adverse birth outcomes (ABO) and is often used to evaluate both national health care systems and racial health disparities [3, 4]. In the United States, infant mortality is commonly grouped into three common causes: birth defects, preterm birth and maternal pregnancy complications. Maternal pregnancy conditions include both severe maternal morbidities (SMM) that occur with labor and delivery and metabolic disorders like gestational diabetes, gestational hypertension and hypertension eclampsia [5]. Each of these common causes of ABO is affected by profound racial health disparities [2, 6].

Despite significant research, the reasons for these racial health disparities remain largely unknown. In their strategic planning, the National Institute on Minority Health and Health Disparities (NIMHD) has long defined a health disparity as a group’s deviation in risk from the overall population risk [7], emphasizing that pair-wise comparisons between two specific sub-populations do not allow for assessment of the overall degree of disparity in an entire population with more than two groups [8, 9]. However the bulk of research into racial health disparities among ABO has concentrated on the risk of race and ethnic groups relative to the specific sub-population of non-Hispanic white women and their children. The objective of this study was to estimate the racial risks among a set of neonatal and maternal health disparities while minimizing bias attributable to how the baseline risk was established. Once racial health disparities are objectively defined and accurately estimated, more specific causal modeling can be addressed. In future research, extending the model that estimated these risks will be able to identify race-specific mediators of race-specific health risks.

## Methods

All birth records were obtained from the United States Natality database for the years 2014 to 2017 [10]. The variables extracted from the database included maternal race and ethnicity, maternal pregnancy conditions, severe maternal morbidities, neonatal conditions, neonatal treatments, birth defects and neonatal death. Maternal race and ethnicity was retained as eight categories: non-Hispanic white, non-Hispanic black, American Indian and Alaska Native (AIAN), Asian, Native Hawaiian and Other Pacific Islanders (NHOPI), Hispanic, mixed and unknown. All eight categories were used in the analysis and referred to as “race.” Estimates for maternal races recorded as “mixed” and “unknown” are not reported. Infants were assigned the maternal race for all births regardless of the paternal race. Maternal pregnancy conditions included gestational diabetes, gestational hypertension and hypertension eclampsia and severe maternal morbidities included induction of labor, caesarian section, maternal transfusion, perineal laceration, ruptured uterus, unplanned hysterectomy and admission to intensive care unit (ICU). Neonatal outcomes included birthweight, gestation length, Apgar score and the occurrence of seizures. Neonatal treatments included antibiotics, administration of surfactant, whether ventilation was started, whether ventilation was prolonged (> 6 h) and whether the child was admitted to a neonatal intensive care (NICU). Very preterm delivery (VPTD) was defined as a gestation period of less than 32 weeks. Preterm delivery (PTD) was defined as gestation period of less than 37 weeks. Large for gestational age (LGA) was defined as the largest 10% of birth weights by gestation length and small for gestational age (SGA) as the smallest 10% of birthweight by gestation length. Birth defects included anencephaly, meningomyelocele/spina bifida, cyanotic congenital heart disease, diaphragmatic hernia, omphalocele, gastroschisis, limb reduction defect, cleft lip (with or without cleft palate), cleft palate alone, hypospadias, Down syndrome and chromosomal disorders. Neonatal death was defined as a death that occurred before the birth certificate was completed. Inclusion criteria are singleton births with all variables of interest available. Of the 15,261,278 singleton births available in the database, 214,036 were excluded due to missing variables, leaving 15,047,242 births to be used in the analysis.

A Bayesian modeling approach was used to estimate the odds ratios for disorders by race. The estimation of the race-specific risks used a sum-to-zero constraint for the adjusted race regression coefficients as follows: Case counts were cross tabulated by *i = 8* race/ethnicities and *j = 34* disorders. For each row in the table, *Y*_*ij*_ was the count of cases at birth and *n*_*i*_ the count of births. The counts (*Y*_*ij*_) were modeled as independent Binomial distributions conditional on an unknown rate parameter (*u*_*ij*_).

$$ {Y}_{ij}\sim Binomial\ \left({u}_{ij},{n}_i\right) $$

The logit of the rate parameter was then provided a vague Normal prior with a mean = 0 and variance = 1000.

$$ Logit\ \left({u}_{ij}\right)\sim N\ \left(0,1000\right) $$

An adjusted rate parameter (*u*′_*ij*_) was estimated constrained to sum to zero by subtracting the race means (${\overline{u}}_i$) from the unconstrained rate parameter at each iteration of the Markov Chain.

$$ u{\hbox{'}}_{ij}={u}_{ij}-{\overline{u}}_i $$

The odds ratio was estimated as the inverse log of the adjusted rate parameter.

$$ {OR}_{ij}={e}^{u{\prime}_{ij}} $$

For the estimate of percent risk increase, the race risk was estimated by the rate parameter (*u*_*ij*_) and the expected rate was estimated from the race mean parameter (${\overline{u}}_i$). *P*-values were defined as the posterior probability of the percent increase being greater than zero and was taken directly from the full posterior distribution [11]. The implementation used Markov Chain Monte Carlo (MCMC) and the software OpenBUGS 3.2.3 [12]. One thousand iterations were allowed for burn-in and the next 10,000 iterations were collected for the posterior distribution. Monitoring chains for the adjusted rate parameter and the race means was used to determine convergence. The code for analyses and data are available in additional files.

## Results

For all results, the percent increase in risk, Bayesian credibility intervals and *p*-values are presented in Table 1.

**Table 1:** Racial health disparities. The percent increase in risk and 95% credibility intervals for racial health disparities defined as deviations from the population expected rates

	White	Black	AIAN^1^	Asian	NHOPI^2^	Hispanic
***Number of singleton births (n)***	5,680,489	1,513,616	87,318	690,936	25,279	2,568,435
***Maternal conditions***						
Gestational Diabetes	− 23.7 (−24.3, −23.1)	−30.5 (−31.1, − 29.8)	30.3 (27.8, 32.9) ^a^	62.0 (60.5, 63.5) ^a^	23.7 (19.4, 28.1) ^a^	−6.0 (−6.7, −5.2)
Gestational hypertension	10.1 (9.1, 11.1)^a^	28.3 (27.0, 29.5) ^a^	34.9 (32.0, 37.8) ^a^	− 40.4 (− 41.2, − 39.6)	6.2 (1.7, 10.9) ^a^	−14.6 (− 15.4, − 13.7)
Hypertension eclampsia	−21.7 (− 24.5, − 18.9)	32.3 (27.3, 37.5) ^a^	38.1 (25.5, 51.9) ^a^	−42.6 (− 46.0, − 39.2)	175.4 (142.7, 211) ^a^	−37.2 (− 39.6, − 34.5)
***Severe maternal morbidities***						
Induction of labor	25.6 (25.1, 26.1) ^a^	6.0 (5.5, 6.5) ^a^	15.0 (13.8, 16.2) ^a^	−12.7 (− 13.2, − 12.2)	− 18.5 (− 20.3, − 16.7)	−8.7 (− 9.1, − 8.3)
Caesarian section	−2.4 (− 2.7, − 2.1)	14.1 (13.7, 14.5) ^a^	−10.1 (− 11.0, − 9.2)	5.8 (5.4, 6.3) ^a^	0.0 (− 1.6, 1.7)	1.7 (1.3, 2.0) ^a^
Maternal transfusion	−20.1 (− 22.7, − 17.3)	3.1 (− 0.7, 7.2)	115.1 (100.0, 131.2) ^a^	− 27.2 (− 30.9, − 23.4)	26.7 (7.3, 48.4) ^a^	−18.2 (− 21.2, − 15.1)
Perineal Laceration	18.2 (15.3, 21.2) ^a^	−47.5 (− 49.2, − 45.9)	− 17.8 (− 24.0, − 11.2)	116.2 (110.1, 122.5)	10.0 (−2.8, 23.7)	− 25.5 (− 27.5, − 23.5)
Ruptured uterus	−15.3 (− 24.7, − 3.8)	11.7 (−2.4, 28.1)	42.9 (5.2, 90.4) ^a^	−10.3 (− 24.2, 6.0)	−1.6 (− 51.1, 70.8)	−17.9 (− 27.7, − 5.8)
Unplanned hysterectomy	−30.0 (− 35.9, − 23.1)	−14.0 (− 22.5, − 4.2)	64.3 (31.6, 103.5) ^a^	−6.3 (− 17.2, 6.6)	27.8 (− 19.7, 92.4)	−14.6 (− 22.2, − 5.7)
Admission to ICU	−34.8 (− 37.9, − 31.5)	19.4 (13.1, 25.9) ^a^	7.1 (−7.1, 23.4)	− 8.2 (− 14.2, − 1.7)	98.3 (62.5, 137.5) ^a^	−24.7 (− 28.5, − 20.6)
***Birth defects***						
Anencephaly	56.5 (19.8, 136.1) ^a^	5.7 (−23.1, 64.9)	70.8 (−13.6, 212.6)	− 25.5 (− 50.8, 20.3)	− 53.2 (− 96.9, 89.9)	55.1 (16.7, 134.2) ^a^
Meningomyelocele/spina bifida	47.5 (21.3, 91.5) ^a^	−12.3 (− 31.3, 17.7)	118.8 (39.9, 236.1) ^a^	− 59.1 (− 72.1, − 40.2)	−34.2 (− 87.3, 83.9)	12.1 (− 10.2, 47.3)
Cyanotic congenital heart disease	6.5 (0.0, 14.9) ^a^	−21.7 (− 28.6, − 13.8)	45.3 (17.5, 77.6) ^a^	−46.4 (− 53.1, − 39.1)	72.4 (20.5, 136.5) ^a^	−32.6 (− 38.2, − 26.5)
Diaphragmatic hernia	31.2 (9.6, 61.6) ^a^	− 13.2 (− 30.8, 10.8)	36.4 (− 21.3, 120.9)	−33.9 (− 51.7, − 10.9)	38.4 (− 52.0, 199.6)	− 14.1 (− 30.2, 7.7)
Omphalocele	28.7 (3.4, 69.2) ^a^	23.8 (− 3.2, 65.2)	67.5 (− 5.3, 176.7)	− 39.4 (− 57.6, − 11.4)	− 21.5 (− 84.8, 121.5)	−21.2 (− 38.4, 5.4)
Gastroschisis	20.5 (5.0, 41.1) ^a^	−21.4 (− 33.4, − 5.2)	211.6 (141.0, 302.3) ^a^	− 73.2 (− 80.4, − 64.3)	−18.7 (− 68.0, 60.2)	14.5 (− 1.3, 35.8)
Limb reduction defect	19.9 (0.5, 48.2) ^a^	1.5 (− 17.5, 27.7)	152.3 (73.6, 262.2) ^a^	−41.5 (− 56.5, − 20.6)	13.5 (− 61.0, 140.5)	− 15.6 (− 31.0, 5.3)
Cleft lip (with or without cleft palate)	17.1 (6.8, 29.2) ^a^	−37.0 (− 43.9, − 28.9)	117.8 (79.1, 164.8) ^a^	− 22.6 (− 32.7, − 11.0)	−9.4 (− 48.0, 42.7)	−0.4 (− 9.9, 10.8)
Cleft palate alone	26.4 (10.6, 47.9) ^a^	−32.8 (− 43.4, − 19.2)	115.0 (58.7, 187.5) ^a^	−21.7 (− 36.1, − 3.2)	− 17.4 (− 67.5, 65.9)	− 29.8 (− 39.9, − 16.6)
Hypospadias	81.2 (63.2, 104.9) ^a^	10.3 (−2.5, 26.1)	−13.2 (− 37.6, 17.8)	− 6.6 (− 19.5, 8.9)	−28.8 (− 64.2, 24.4)	− 25.3 (− 33.7, − 14.5)
Down syndrome	17.0 (6.3, 30.5) ^a^	−18.6 (− 27.6, − 8.0)	43.4 (11.9, 80.2) ^a^	− 36.5 (− 45.6, − 25.9)	−29.0 (− 62.7, 19.2)	22.5 (10.7, 37.0) ^a^
Chromosomal disorder	8.9 (0.0, 20.9) ^a^	−13.2 (− 23.1, − 1.6)	54.3 (18.7, 97.6) ^a^	− 26.9 (− 37.5, − 14.4)	41.7 (− 14.3, 117.7)	−0.4 (− 10.6, 11.5)
***Neonatal conditions***						
Low Apgar	−21.2 (− 23.4, − 18.9)	78.3 (73.2, 83.8) ^a^	23.1 (14.1, 32.5) ^a^	− 42.2 (− 44.7, − 39.6)	12.9 (−2.2, 29.3)	− 26.3 (− 28.5, − 23.9)
Small for Gestational age	− 22.4 (− 22.9, − 21.9)	51.0 (50.0, 52.0) ^a^	− 24.3 (− 25.8, − 22.7)	32.6 (31.6, 33.7) ^a^	− 10.4 (− 13.2, − 7.4)	−9.1 (− 9.7, − 8.5)
Large for Gestational age	24.9 (24.2, 25.7) ^a^	−36.0 (− 36.5, − 35.5)	50.9 (48.7, 53.2) ^a^	− 41.8 (− 42.4, − 41.2)	39.5 (35.7, 43.3) ^a^	− 1.1 (− 1.7, − 0.5)
Very pre-term delivery	−31.9 (− 33.2, − 30.5)	94.6 (90.8, 98.5) ^a^	1.3 (− 4.0, 7.0)	− 30.5 (− 32.4, − 28.6)	5.2 (− 4.6, 15.4)	−10.3 (− 12.1, − 8.4)
Pre-term delivery	− 18.0 (− 18.6, − 17.4)	34.4 (33.3, 35.4) ^a^	13.9 (11.8, 16.0) ^a^	−18.0 (− 18.9, − 17.2)	11.8 (8.1, 15.6) ^a^	− 6.1 (− 6.8, − 5.4)
Seizures	22.9 (9.1, 40.7) ^a^	12.2 (− 1.7, 30.5)	59.6 (19.3, 109.2) ^a^	− 27.0 (− 38.8, − 12.0)	− 28.9 (− 68.7, 31.7)	−34.3 (− 42.5, − 23.9)
***Neonatal treatments***						
Antibiotics	−6.8 (− 8.2, − 5.4)	15.0 (13.2, 16.9) ^a^	24.7 (20.2, 29.3) ^a^	− 6.8 (− 8.6, − 4.9)	− 6.2 (− 13.1, 1.3)	−13.1 (− 14.5, − 11.7)
Surfactant	6.3 (2.6, 10.4) ^a^	53.6 (47.8, 59.8) ^a^	15.4 (4.6, 26.7) ^a^	−43.2 (− 46.4, − 39.9)	2.8 (− 15.1, 22.0)	− 18.2 (− 21.4, − 14.8)
Begin ventilation	5.1 (4.0, 6.2) ^a^	15.4 (14.1, 16.7) ^a^	26.3 (22.8, 29.8) ^a^	− 32.2 (− 33.2, − 31.1)	38.3 (31.9, 44.9) ^a^	−27.8 (− 28.7, − 27.0)
Prolonged ventilation (> 6 h)	0.4 (− 1.4, 2.3)	25.0 (22.5, 27.6) ^a^	24.7 (18.7, 30.9) ^a^	−36.5 (− 38.3, − 34.6)	31.9 (20.7, 43.7) ^a^	−27.0 (− 28.4, − 25.4)
Admission to NICU	−13.1 (− 13.7, − 12.5)	26.8 (25.8, 27.8) ^a^	0.9 (− 1.1, 3.1)	− 10.0 (− 10.9, − 9.1)	5.0 (1.2, 8.8) ^a^	− 7.6 (− 8.3, − 6.9)
***Neonatal mortality***	−38.2 (− 40.1, − 36.2)	9.1 (5.4, 12.9) ^a^	−2.1 (− 10.9, 7.4)	− 25.6 (− 29.0, − 22.2)	41.5 (22.8, 62.9) ^a^	− 8.8 (− 11.8, − 5.8)

^a^ Statistically significant disparity (*p* < 0.05)

White non-Hispanic women had increased risk for developing gestational hypertension, having a caesarian section and experiencing a perineal laceration. Children of these women had increased risk for LGA, having seizures and had increased risk for 10 monitored birth defects and both Down syndrome and chromosomal disorders.

Women who were classified as black and non-Hispanic were more likely to develop gestational hypertension and hypertension eclampsia. Increased risks for SMM included induction of labor, caesarian section and admission to the ICU. Their children had increased risk for VPTD, PTD and SGA as well as low Apgar scores. They were more likely to be treated with antibiotics and surfactant and more likely to be started on ventilation, to have received prolonged ventilation and more likely to have been admitted to the NICU.

American Indian and Alaska Native (AIAN) women had increased risk for developing gestational diabetes, gestational hypertension and hypertension eclampsia. Increased risk for SMM included induction of labor, maternal transfusion and admission to ICU. Neonatal risks were increased for LGA, PTD, low Apgar and seizures. These children had increased risk for birth defects, including meningomyelocele/spina bifida, cyanotic congenital heart disease, gastroschisis, limb reduction defect, cleft lip, cleft palate as well as for Down syndrome and chromosomal disorders. Increased risks for neonatal treatments included treatment with antibiotics, surfactant, beginning ventilation, prolonged ventilation and admission to NICU.

Native Hawaiian and Other Pacific Islanders (NHOPI) women had increased risk of developing gestational diabetes, hypertension and hypertension eclampsia. Increased risks for SMM included for maternal transfusion and admission to ICU. Children of NHOPI women had increased risk of PTD and LGA and the group of birth defects referred to as cyanotic congenital heart disease. These children were more likely to have been started on ventilation, received prolonged ventilation and to have been admitted to NICU.

Asian and Hispanic women were relatively free of ABO. The exceptions included that Asian women had increased risk of developing gestational diabetes and had increased risk for caesarian section or perineal laceration. Asian children had no increased risks for the ABO studied. Hispanic women had a very small elevation of risk for caesarian section and their children had increased risk for Down syndrome and anencephaly.

## Discussion

For the purpose of the current study, the authors define “disparity” as any increase in risk for ABO based on an individual’s race or ethnicity when compared to the overall population risk rate. The NIMHD cites a colorful debate regarding the use of the word “disparity” but clarify, for the sake of strategic planning, that racial health disparity means any increase regardless of whether the difference seems unjust with “unjust” meaning attributable to a policy that affects health inequitably among races [7]. This approach is most appropriate because causal modeling must be relatively well defined before a disease cause can be debated as “just or unjust.” For example, the Bayesian patient-based model of the current study could be expanded to include hierarchal variables that could demonstrate risks of social policies that would satisfy most criteria for an “unjust” cause. The NIMHD in providing this working definition is stipulating that the risks should be estimated for each group relative to the overall population risk. The objective of the current study was to estimate racial disparity risk for 34 ABOs among 15 million births in the United States over a recent four-year period. In spite of the NIMHD working definition for disparity, there is a large body of literature describing racial health disparities comparing the disparity to the specific sub-population of non-Hispanic white women and their children. This is true for pregnancy conditions and SMM [5], birth defects [13–15] and neonatal morbidities [2, 16, 17]. The current modeling was performed using a Bayesian approach and a readily available software package and by using a sum-to-zero constraint for the model coefficients [18]. This MCMC implementation maintains the favorable Bayesian properties of full posterior distributions for all of the parameters estimated. Point estimates of the pairwise comparisons can be derived from results of the current study from the ratio of the median of the two odds ratios. Furthermore, with the data and model available in the additional files, the full posterior distribution of any pairwise comparison can be estimated.

Much of the existing literature that describes racial disparities adjusts the disparity risk estimate for covariates like socioeconomic status (SES), for example [2, 5, 9, 13–17]. The rationale for conditioning on covariates has not always been clear. Following a unifying and serviceable definition for confounding [19], there are no backdoor paths between race and any of the outcomes in the current study because none of the factors can “cause” race. Backdoor paths for the more abstract construct “racism” can be envisioned and confounding control defended in those models [4] but controlling for confounding by racism was not an objective of the study and should not be performed for descriptions of potential racial disparities. For the unchangeable factor of race, any covariates used for conditioning should be considered potential mediators and it is the effect of the mediator in changing the risk estimate for each race that is of interest, not just the resultant conditional risk [20]. Future work should evaluate specific mediation models and effects but should start with the best possible estimates for the risk of racial health disparity.

The current study yielded novel risk estimates for white non-Hispanic women and their children. These racial health disparities should be considered potentially comorbid conditions. Among non-Hispanic women and their children, Down syndrome is known to be associated with comorbidities including birth defects, seizures and multiple other conditions that can lead neonatal death [21]. In another causal web, LGA, gestational hypertension, and either caesarian section or perineal laceration are known to be associated with both gestational weight gain and pre-pregnancy Body Mass Index [22]. While induction of labor and caesarian deliveries are not considered severe compared to other maternal morbidities studied, they have been included as a maternal morbidity. However, induction of labor or caesarian section likely contributes to avoidance of less favorable maternal and neonatal outcomes and because they often increase the number of good outcomes, they should not be automatically classified as SMM. A limitation of this study is that the database does not identify indication for cesarean delivery. Furthermore, information on whether PTD was indicated or spontaneous is also not available. Further research on how these associate with health disparities is needed. It is possible that the racial disparities among caesarian delivery and labor induction may be explained by comorbid conditions.

Changing the baseline rate to the population rate rather than the rate among non-Hispanic white women and their children lowered the estimated risk rate for many of the ABO for non-Hispanic black women and their children. This results because white non-Hispanic women and their children have lower than population average risk for multiple ABO. This was especially notable among VPTD, PTD and SGA and a well-known wide range of comorbidities, including neonatal mortality. In spite of the lowered risk estimates, the list of comorbidities remains relatively unchanged and included pregnancy complications, SMM, neonatal morbidity and neonatal treatments. These disparities were both statistically and clinically significant and the need further investigation into the causes continues to be urgent.

The current results estimate a wide range of comorbidities for AIAN women and their children. Included among the comorbidities are five birth defects for which the risk for children of AIAN women are at least double the risk rate of the overall population. These birth defects include meningomyelocele/spina bifida; gastroschisis; limb reduction defect; cleft lip and cleft palate.

Women classified as NHOPI had increased risk of pregnancy complications including a more than doubling of risk for hypertension eclampsia and a near doubling of risk for admission to ICU. These two conditions are considered to be causally linked [23] and are jointly caused by maternal obesity, which is more prevalent among NHOPI women [24]. The children of NHOPI women had increased risk of cyanotic congenital heart disease, LGA and PTD and to be treated with short- and long-term ventilation and admission to NICU. In addition to causing maternal conditions, maternal obesity is also associated with both LGA and PTD [21]. This cluster of comorbidity may also include a causal connection between maternal obesity and cyanotic congenital heart disease [25–27] and the treatment sequelae [28]. Racial health disparities for birth defects among children of NHOPI women have been reported but the risks were estimated relative to non-Hispanic white women and their children and the risk was adjusted for maternal age without a reporting of the mediation effect of maternal age [13, 14]. More study is needed to evaluate the large cluster of potentially causal associations.

The authors of a recent special journal edition claimed that what were needed most following a refocusing definition were analytic methods addressing the ability to draw causal inferences from observational studies [8, 29]. We anticipate that the ultimate causal explanations will build on the concept that multiple disorders within a racial, ethnic or otherwise defined disadvantaged group are comorbidities and share common causal elements. All of these causal elements will be downstream from race and thus should be considered potential mediators of the effects of racial health disparity but not confounders. How the comorbidities fit into a causal web as mediators will present a vexing problem. The list of comorbidities is relatively well defined, however, the causal ordering of common causal factors among the many pregnancy complications, SMM, neonatal morbidity and neonatal treatments will always be debatable. However, causal modeling is encouraged and reports should include the causal modeling assumptions which will enable the long-term goal of identifying causes of racial disparities that can be addressed.

## Conclusions

Estimating racial health disparities relative to the overall population expected rate yielded novel results and identified perinatal health disparities for all the race and ethnic groups studied. Unbiased risk estimates for racial and ethnic disparities among ABO should be used to initiate more complex causal modeling that can lead to understanding how racial health disparities for ABO are mediated and how they can be prevented.

## Supplementary information

**Additional file 1.** WinBUGS code and data. This code and these data can be used to repeat the analyses reported here.

## Data Availability

The database used in the study is publically available (https://www.cdc.gov/nchs/data_access/vitalstatsonline.htm). These data collected by the National Center for Health Statistics may be used only for the purpose of health statistical reporting and analysis. The authors consented a data use agreement (https://www.cdc.gov/nchs/data_access/restrictions.htm). The tabulated data and the analysis code for OpenBUGS are available in Additional file 1.

## Abbreviations

AIAN: American Indian and Alaska Native
ICU: Intensive care unit
IRB: Institutional review board
LGA: Large for gestational age
MCMC: Markov Chain Monte Carlo
NHOPI: Native Hawaiian and other Pacific Islanders
NICU: Neonatal intensive care unit
PTD: Preterm delivery
SES: Socio-economic status
SGA: Small for gestational age
SMM: Severe maternal morbidity
VPTD: Very preterm delivery
