# Supplementary material for: Estimating racial health disparities among adverse birth outcomes as deviations from the population rates
Source: BMC Pregnancy Childbirth. 2020 Mar 12;20:155. doi: 10.1186/s12884-020-2847-9 (PMC7069047; doi:10.1186/s12884-020-2847-9)
Supplement: Supplementary file 1 — Additional file 1. WinBUGS code and data. This code and these data can be used to repeat the analyses reported here. [file 12884_2020_2847_MOESM1_ESM.docx]

**Additional Files**

model { for (i in 1:8) { for (j in 1:34) { r[i,j] ~ dbin(prev[i,j], n[i])

logit(prev[i,j]) <- race[i,j]

race[i,j] ~ dnorm(0,0.01)

P[i,j] <- 1 - step(race[i,j] - meanrisk[j])

OR[i,j] <- exp(race[i,j] - meanrisk[j])

PIR[i,j] <- 100*(prev[i,j] - base.prev[j]) / base.prev[j] }}

for (j in 1:34){ meanrisk[j] <- mean(race[ ,j])

base.odds[j] <- exp(meanrisk[j])

base.prev[j] <- base.odds[j] / (base.odds[j] + 1) }}

n[] r[ ,1] r[ ,2] r[ ,3] r[ ,4] r[ ,5] r[ ,6] r[ ,7] r[ ,8] r[ ,9] r[ ,10] r[ ,11] r[ ,12] r[ ,13] r[ ,14] r[ ,15] r[ ,16] r[ ,17] r[ ,18] r[ ,19] r[ ,20] r[ ,21] r[ ,22] r[ ,23] r[ ,24] r[ ,25] r[ ,26] r[ ,27] r[ ,28] r[ ,29] r[ ,30] r[ ,31] r[ ,32] r[ ,33] r[ ,34]

7847394 405376 465143 18126 2194270 2310916 21685 74022 2080 2673 8794 36012 652618 936555 70480 548069 540525 90218 28481 836 1307 5459 1148 886 2292 1215 4778 2291 6126 4483 3248 293505 29348 161485 3149

2128412 99792 147237 8413 501640 728392 7697 8717 784 866 4540 22304 344199 130596 54226 243822 214641 30325 11493 162 205 1066 213 226 394 282 678 312 971 820 683 87285 11409 53175 759

122477 10643 8859 513 31017 33175 947 760 56 96 236 893 10100 17576 1625 11977 9932 1729 589 19 26 112 19 16 92 38 141 64 48 81 68 5737 494 3304 61

945287 102025 29928 1495 181184 300916 2395 15300 264 426 1464 3150 133289 53133 8460 65055 66388 6580 3726 50 47 348 70 60 65 69 381 184 359 291 258 22473 1825 18827 223

36071 2974 2010 240 6400 10837 150 281 12 23 113 240 3489 4787 486 3543 2920 523 226 2 2 34 4 4 9 6 20 6 9 14 17 1773 136 753 13

305392 17046 17249 1095 77179 86756 879 2193 87 102 446 1753 33546 28895 4033 25389 24626 3905 1521 19 45 199 32 34 136 47 141 65 171 157 96 12411 1226 6956 117

3283158 209889 148525 5997 653708 998151 9103 18784 829 1354 4343 13727 310854 314167 37096 256985 233691 25794 15112 347 393 1358 309 222 883 351 1675 508 924 1868 1187 81738 8614 58800 650

122430 7037 6087 201 23231 35768 428 1093 47 85 244 1157 13459 11556 2189 10511 10413 1352 1476 11 15 145 15 8 28 11 60 32 53 78 41 3970 474 2698 56

END

Abbreviations are as described in the materials and methods or as defined in the database description found here: (<https://www.cdc.gov/nchs/data_access/vitalstatsonline.htm>)

order of races 1= white; 2=black; 3=AIAN; 4=Asian; 5=NHOPI; 6=Mixed; 7=Hispanic; 8=unknown;

order of outcomes 1 diab 2 hypertension 3 eclampsia 4 INDL 5 csection 6 MTR 7 PLAC 8 RUPT 9 UHYST 10 AICU 11 apgar 12 SGA 13 LGA 14 VPTD 15 PTD 16 NICU 17 vent6 18 death 19 anen 20 sb 21 cchd 22 cdh 23 omph 24 gast 25 limb 26 cleft 27 clpal 28 hypo 29 DS 30 CD 31 vent1 32 surf 33 anti 34 seiz;
